# Trend analysis and modelling of universal health coverage, Ethiopia

**DOI:** 10.2471/BLT.24.292995

**Published:** 2025-11-03

**Authors:** Yibeltal Assefa, Yalemzewod Assefa Gelaw, Aklilu Endalamaw, Eskinder Wolka, Anteneh Zewdie

**Affiliations:** a School of Public Health, University of Queensland, Herston Road, Brisbane, Queensland, 4006, Australia.; b The Kids Research Institute, Perth, Australia.; c Global Programs, International Institute of Primary Health Care, Addis Ababa, Ethiopia.

## Abstract

**Objective:**

To investigate the attainability of the 2030 universal health coverage (UHC) target of 80% using Ethiopia as a case study.

**Methods:**

We examined trends in Ethiopia’s universal health service coverage index and its subindices between 2000 and 2021 in Ethiopia. To assess long-term progress, we projected coverage to 2030 and 2040 using Bayesian models. Simulation models evaluated the effect of expanding health system inputs, including service capacity and funding, on UHC.

**Findings:**

Ethiopia’s universal health service coverage index increased steadily from 2000 to 2015 and slowed between 2015 and 2019 before progress stalled between 2019 and 2021. Projections indicate the country will achieve a UHC index of 64.7% by 2030, failing to meet the 80% target. Projected subindex values for 2030 were 68.4% for reproductive, maternal, neonatal and child health and 66.1% for infectious diseases but only 58.5% for noncommunicable diseases. Simulation modelling indicated that doubling health system inputs would only modestly increase UHC and that the 80% target is unlikely to be reached before 2040.

**Conclusion:**

The trajectory of UHC in Ethiopia reflects both achievements and persistent gaps in health services. Modelling suggests that boosting health system inputs alone will be insufficient to reach the 80% coverage target by 2030. Structural reforms, better governance and greater system integration are required. The modelling framework used could help other countries assess progress and design context-specific, equitable pathways towards UHC.

## Résumé

**Objectif:**

Étudier la faisabilité de l’objectif de 80% de couverture sanitaire universelle (CSU) d’ici à 2030 en prenant l’Éthiopie comme étude de cas.

**Méthodes:**

Nous avons examiné les tendances de l’indice de couverture sanitaire universelle en Éthiopie et de ses sous-indices entre 2000 et 2021. Afin d’évaluer les progrès à long terme, nous avons projeté la couverture en 2030 et en 2040 à l’aide de modèles bayésiens. Des modèles de simulation ont évalué l’effet, sur la CSU, de l’augmentation des investissements dans le système de santé, notamment la capacité de service et le financement.

**Résultats:**

L’indice de couverture sanitaire universelle en Éthiopie a augmenté continuellement de 2000 à 2015, puis a ralenti entre 2015 et 2019, avant de stagner entre 2019 et 2021. D’après des projections, le pays atteindra un indice de couverture sanitaire universelle de 64,7% d’ici à 2030, ce qui ne permettra pas d’atteindre l’objectif de 80%. Les valeurs prévisionnelles du sous-indice pour 2030 étaient de 68,4% pour la santé reproductive, maternelle, néonatale et infantile et de 66,1% pour les maladies infectieuses, mais de seulement 58,5% pour les maladies non transmissibles. La modélisation par simulation a indiqué que le doublement des investissements dans le système de santé n'augmentera que modestement la couverture sanitaire universelle et que l’objectif de 80% ne serait probablement pas atteint avant 2040.

**Conclusion:**

La trajectoire de la CSU en Éthiopie reflète à la fois les progrès accomplis et les lacunes persistantes dans les services de santé. La modélisation suggère que la simple augmentation des investissements dans le système de santé ne suffira pas pour atteindre l’objectif de couverture de 80% d’ici à 2030. Des réformes structurelles, une meilleure gouvernance et une plus grande intégration du système sont nécessaires. Le cadre de modélisation employé permettrait à d’autres pays d’évaluer les progrès accomplis et de concevoir des trajectoires équitables et adaptées à leur contexte pour parvenir à la CSU.

## Resumen

**Objetivo:**

Investigar la posibilidad de alcanzar para 2030 la meta del 80% de cobertura sanitaria universal (CSU), utilizando Etiopía como estudio de caso.

**Métodos:**

Se examinaron las tendencias del índice de cobertura de servicios sanitarios universales de Etiopía y de sus subíndices entre 2000 y 2021. Para evaluar los avances a largo plazo, se proyectó la cobertura hasta 2030 y 2040 mediante modelos bayesianos. Asimismo, se utilizaron modelos de simulación para evaluar el efecto de ampliar los insumos del sistema de salud, incluida la capacidad de prestación de servicios y la financiación, sobre la CSU.

**Resultados:**

El índice de cobertura de servicios sanitarios universales de Etiopía aumentó de forma constante entre 2000 y 2015, se desaceleró entre 2015 y 2019 y se estancó entre 2019 y 2021. Las proyecciones indican que el país alcanzará un índice de CSU del 64,7% en 2030, sin llegar al objetivo del 80%. Los valores proyectados de los subíndices para 2030 fueron del 68,4% para salud reproductiva, materna, neonatal e infantil; del 66,1% para enfermedades infecciosas; y solo del 58,5% para enfermedades no transmisibles. La modelización mediante simulación mostró que duplicar los insumos del sistema de salud incrementaría la CSU solo de manera modesta y que es improbable alcanzar el objetivo del 80% antes de 2040.

**Conclusión:**

La trayectoria de la CSU en Etiopía refleja tanto los logros alcanzados como las deficiencias persistentes en los servicios de salud. La modelización sugiere que el aumento de los insumos del sistema de salud, por sí solo, no será suficiente para alcanzar la meta del 80% de cobertura en 2030. Se requieren reformas estructurales, una mejor gobernanza y una mayor integración del sistema. El marco de modelización empleado podría servir a otros países para evaluar sus progresos y diseñar trayectorias equitativas y adaptadas a sus contextos hacia la CSU.

## ملخص

الغرض النظر في إمكانية تحقيق هدف التغطية الصحية الشاملة (UHC) لعام 2030، وهو %80، مع الإشارة لإثيوبيا كدراسة حالة.

**الطريقة:**

قمنا بفحص اتجاهات مؤشر تغطية الخدمة الصحية الشاملة في إثيوبيا ومؤشراته الفرعية بين عامي 2000 و2021. لتقييم مدى التقدم على المدى الطويل، توقعنا حجم التغطية بين عامي 2030 و2040 باستخدام نماذج Bayesian. قامت نماذج المحاكاة بتقييم أثر تمديد مدخلات النظام الصحي على التغطية الصحية الشاملة، بما في ذلك القدرة الاستيعابية للخدمات والتمويل.

النتائج إن مؤشر تغطية الخدمة الصحية الشاملة في إثيوبيا قد ارتفع بشكل مطرد بين عامي 2000 و2015، وتباطأ بين عامي 2015 و2019 قبل أن يتوقف التقدم بين عامي 2019 و2021. تشير التوقعات إلى أن الدولة ستحقق مؤشر تغطية صحية شاملة بنسبة %64.7 بحلول عام 2030، بالإخفاق عن تحقيق هدف الـ %80. بلغت قيم المؤشرات الفرعية المتوقعة لعام 2030 نسبة %68.4 للصحة الإنجابية، وصحة الأم، والمولود، والطفل، و%66.1 للأمراض المعدية، و%58.5 فقط للأمراض غير المعدية. وأوضح وضع نماذج المحاكاة أن مضاعفة مدخلات النظام الصحي لن تزيد التغطية الصحية الشاملة إلا بشكل طفيف، وأن هدف الـ %80 من غير المرجح أن يتحقق قبل عام 2040.

الاستنتاج يعكس مسار التغطية الصحية الشاملة في إثيوبيا كلاً من الإنجازات والفجوات المستمرة في الخدمات الصحية. ويقترح وضع النماذج إلى أن تعزيز مدخلات النظام الصحي وحده لن يكون كافياً لتحقيق هدف التغطية بنسبة الـ %80 بحلول عام 2030. ويتطلب الأمر إصلاحات هيكلية، وحوكمة أفضل، وتكاملاً أكبر للنظام. ويمكن لإطار وضع النماذج المستخدم أن يساعد الدول الأخرى على تقييم التقدم، وتصميم مسارات عادلة ومحددة السياق، نحو التغطية الصحية الشاملة.

## 摘要

**目的:**

基于埃塞俄比亚开展案例研究，以探讨 2030 年实现 80% 全民健康覆盖 (UHC) 率这一目标的可能性**。**

**方法:**

我们研究了埃塞俄比亚在 2000 年至 2021 年期间的全民健康服务覆盖指数及其分指数的发展趋势。为了评估长期进展，我们运用贝叶斯模型预测了截至 2030 年和 2040 年的覆盖率。我们运用模拟模型评估了加大卫生系统投入（包括服务能力和资金）会对 UHC 率产生的影响。

**结果:**

埃塞俄比亚的全民健康服务覆盖指数在 2000 年至 2015 年期间稳定增长，在 2015 年至 2019 年期间增长速度有所下降，而在 2019 年至 2021 年期间却毫无增长。预测结果表明，截至 2030 年，埃塞俄比亚的 UHC 指数将达到 64.7%，即无法实现 80% 的目标值。据预测，截至 2030 年，该国生殖、孕产妇、新生儿和儿童健康领域的分指数值为 68.4%，传染病领域的分指数值为 66.1%，但非传染性疾病领域的分指数值却仅为 58.5%。模拟建模结果表明，即使将卫生系统投入加大一倍，也只能适当提高 UHC 率，却仍无法在 2040 年之前实现覆盖率达到 80% 这一目标。

**结论:**

埃塞俄比亚的 UHC 率发展轨迹反映了其在卫生服务方面所取得的成就以及持续存在的不足之处。建模结果表明，仅加大卫生系统投入并不足以助力该国在 2030 年之前实现覆盖率达到 80% 这一目标。如要实现这一目标，还需推动结构改革、完善管理并加强系统集成。所采用的建模框架可助力其他国家评估其 UHC 率发展情况并设计符合本国具体国情且公平合理的 UHC 率实现途径。

## Резюме

**Цель:**

На примере Эфиопии изучить, насколько может быть реально к 2030 году достичь 80%-го целевого показателя всеобщего охвата услугами здравоохранения (ВОУЗ).

**Методы:**

Авторы изучили тенденции изменения показателя всеобщего охвата услугами здравоохранения и его различных составляющих для Эфиопии в период между 2000 и 2021 годами. Для оценки долгосрочного прогресса авторы спрогнозировали охват до 2030 и 2040 годов с помощью байесовских моделей. Имитационное моделирование позволило оценить эффект расширения вложений в систему здравоохранения, включая возможности оказания услуг и их финансирование, на достижение ВОУЗ.

**Результаты:**

Показатель всеобщего охвата услугами здравоохранения в Эфиопии неуклонно рос в период с 2000 по 2015 год, замедлился в период с 2015 по 2019 год, в конечном итоге прогресс остановился в период с 2019 по 2021 год. Прогнозы показывают, что к 2030 году страна достигнет уровня ВОУЗ в 64,7%, что не соответствует целевому показателю в 80%. Прогнозируемые значения по отдельным составляющим на 2030 год были таковы: 68,4% для репродуктивной медицины, охраны здоровья матерей, новорожденных и детей, 66,1% для инфекционных заболеваний и всего лишь 58,5% для неинфекционных заболеваний. Результаты имитационного моделирования указывают на то, что удвоение вложений в систему здравоохранения приведет лишь к незначительному увеличению показателя ВОУЗ и что страна вряд ли сможет достичь 80%-го целевого охвата до 2040 года.

**Вывод:**

Траектория изменения показателя ВОУЗ в Эфиопии отражает как достижения, так и сохраняющиеся пробелы в системе здравоохранения. Моделирование позволяет предположить, что одного лишь увеличения вложений в систему здравоохранения будет недостаточно для достижения 80%-го охвата к 2030 году. Требуются структурные реформы, улучшение управления и усиление системной интеграции. Использованная рамочная схема моделирования может помочь другим странам в оценке их прогресса и создании равных по возможностям и учитывающих местные условия путей достижения цели ВОУЗ.

## Introduction

Universal health coverage (UHC) is a fundamental goal of health systems worldwide, and ensures that all individuals receive health services according to their needs without experiencing financial hardship.^1^ There are two components of UHC: effective service coverage and financial protection.^2^ The World Health Organization (WHO) included UHC as a core health system objective in its 2018 monitoring and evaluation framework for primary health care.^3^^,^^4^ UHC is monitored using 14 tracer indicators grouped into four categories: (i) maternal, reproductive, neonatal and child health; (ii) infectious diseases; (iii) noncommunicable diseases; and (iv) service capacity and access.^5^ Indicators are selected on the basis of the burden of diseases and treatment availability, cost–effectiveness and measurability, with clear numerators and denominators.^6^^,^^7^ A universal health service coverage index derived from these indicators provides a measure of essential services and it is the aim of every country to achieve coverage of 80% or higher by 2030.^2^

In 2021, around 4.5 billion people out of a global population of 7.9 billion did not have full access to essential health services.^8^ This disparity might have been caused by disruption in services due to the coronavirus disease 2019 (COVID-19) pandemic, rising health-care costs or other obstacles rooted in the community and health systems that stem, for example, from ineffective health system leadership, an unevenly distributed health workforce, weak financial administration or poor infrastructure.^9^^,^^10^

Ethiopia presents a compelling case study of a country that is striving to meet UHC targets. Over the past two decades, the country has made substantial investments in its health system. In 2016, the government launched the Health Sector Transformation Plan, which articulated Ethiopia’s vision for achieving UHC.^11^ Subsequently, the health ministry developed a comprehensive package of essential health services in 2019.^12^ Today, community health programmes led by health extension workers play a critical role in promoting health literacy, disease prevention and access to care in underserved areas.^13^ In addition, Ethiopia has expanded its health-care infrastructure and established thousands of health posts, health centres and hospitals across the country.^13^ Although the number of health workers has also been increased, there remain gaps in their distribution, particularly in rural areas.^14^ Health finance in Ethiopia relies on government funding, donor assistance and household out-of-pocket expenditure.^15^

Although a 2015 study showed that Ethiopia has made clear progress towards UHC,^16^ substantial inequalities in access and variations in the quality of care persist, which impede progress.^17^ Hence, whether the country will achieve the 80% service coverage target by 2030 is still uncertain. Research into health services in Ethiopia, including trend analysis and forecasting, would help guide the development of the health systems and policies required to achieve UHC.^18^

The aim of this study was to investigate the attainability of UHC by analysing trends in the universal health service coverage index and projecting future trajectories, using Ethiopia as a case study. Our findings offer both insights into Ethiopia’s decision-making processes and provide an analytical framework that other countries can apply to evaluate, and accelerate, progress towards UHC.

## Methods

The study involved three approaches: (i) a descriptive and trend analysis; (ii) Bayesian projection modelling; and (iii) simulating the universal health service coverage index and its subindices. These subindices include reproductive, maternal, neonatal and child health; infectious diseases; noncommunicable diseases; and health service capacity and access. Projections of the universal health service coverage index were extended to 2040 to assess long-term trends under various scenarios.

### Universal health service coverage index

The universal health service coverage index is a composite of 14 indicators grouped into four categories.^19^ The reproductive, maternal, neonatal and child health category includes four indicators: (i) family planning; (ii) antenatal care (more than four visits); (iii) diphtheria, tetanus toxoid and pertussis (DTP3) immunization; and (iv) care-seeking for acute respiratory illness. The infectious disease category includes four indicators: (i) effective tuberculosis treatment; (ii) antiretroviral treatment (ART) coverage; (iii) access to insecticide-treated bed nets; and (iv) water, sanitation and hygiene. The noncommunicable disease category includes three indicators: (i) hypertension treatment; (ii) diabetes prevalence; and (iii) the non-use of tobacco. The health service capacity and access category includes three indicators: (i) hospital bed density; (ii) health worker density; and (iii) the International Health Regulations (2005) core capacity index.^20^

### Data sources

To ensure data were comprehensive, we derived values for each indicator from several publicly available sources, including WHO, the World Bank, Our World in Data and national health reports (Table 1).^21^ National health data came from national health surveys conducted in Ethiopia between 2000 and 2021, and we obtained data on health service coverage (e.g. the number of health centres per 100 000 population) for the same time period from Ethiopia’s routine coverage reports submitted to WHO.^22^ Information from WHO included data on UHC indicators and estimates of coverage for reproductive, maternal, newborn and child health, infectious diseases, noncommunicable diseases, and health service capacity and access. Data on the share of health-care expenditure derived from out-of-pocket contributions were obtained from WHO’s Global Health Observatory database.^22^ Detailed descriptions of the indicators and of index calculations are available in the WHO and World Bank’s tracking UHC 2023 global monitoring report.^2^

**Table 1:** Health service coverage parameters, achieving universal health coverage by 2030, Ethiopia, 2000–2021

Health service coverage parameter	Derivation	Data source
Composite universal health service coverage index	Derived from: (i) the reproductive, maternal, neonatal and child health subindex; (ii) the infectious disease subindex; (iii) the noncommunicable disease subindex; and (iv) the health service capacity and access subindex	NA
Reproductive, maternal, neonatal and child health subindex	Derived from: (i) a family planning measure; (ii) the proportion of pregnant women who had ≥ 4 antenatal care visits; (iii) the DTP3 immunization rate; and (iv) a measure of care-seeking for suspected acute respiratory illness	WHO Global Health Observatory and Our World in Data
Infectious disease subindex	Derived from: (i) the tuberculosis treatment success rate as a percentage of new cases; (ii) ART coverage as a percentage of people living with an HIV infection; (iii) a water, sanitation and hygiene measure; and (iv) an insecticide-treated bed nets measure	World Bank Group and Our World in Data
Noncommunicable disease subindex	Derived from: (i) a measure of treatment for hypertension; (ii) diabetes prevalence; and (iii) a measure of tobacco non-use	Our World in Data, WHO Global Health Observatory and World Bank Group
Service capacity and access subindex	Derived from: (i) the hospital bed density; (ii) health worker density; and (iii) the International Health Regulations (2005) core capacity index^20^	WHO Global Health Observatory
Health centres per 100 000 population	The number of health centres in the country divided by the population	WHO Global Health Observatory
Health expenditure per capita	The average amount of money spent on health per person in a country	World Bank Group

ART: antiretroviral therapy; DTP: diphtheria–tetanus–pertussis; HIV: human immunodeficiency virus; NA: not applicable; WHO: World Health Organization.

### Data analysis

We conducted a trend analysis of Ethiopia’s universal health service coverage index between 2000 and 2021 and projected its future trajectory to 2040. Additionally, we used simulation models to explore how changes in key health system inputs could impact service coverage.

#### Descriptive and trend analysis

We tracked changes in health service coverage over time and identified overall trends and improvements in each subindex of the universal health service coverage index. We calculated the average annual growth rate in coverage for each subindex and for the composite universal health service coverage index, which enabled us to quantify the pace of improvement of key health metrics and to identify areas in Ethiopia’s health system where progress was rapid or which persistently underperformed.

#### Bayesian projection modelling

We employed a Bayesian linear regression model with noninformative priors, which provide a neutral starting point, as outlined in previous studies.^23^^,^^24^ The model was used to estimate trends in the universal health service coverage index in Ethiopia over time and to derive the posterior predictive probability distribution of the composite estimated index. The model specified a sampling distribution for the data and a prior distribution for the regression coefficients. The cumulative indices proportions were logit-transformed before analysis, and the transformed values were used in the model before being transformed back to probabilities, thereby ensuring that all predicted and projected probabilities remained between 0 and 1. Posterior parameters were estimated using a Markov Chain Monte Carlo simulation with Gibbs sampling, as employed by R2jags version 0.8.5 (The R Foundation, Vienna, Austria).^25^ After drawing an initial 1000 samples from the posterior distributions, the first 5000 iterations were discarded during the burn-in phase of the simulation and sampling was continued until convergence was apparent. These posterior predictive distributions were used to generate projections and 95% credible intervals (CrIs) up to the year 2030 or 2040, as appropriate.

#### Simulation model

We developed a simulation model to assess the impact of changes in three key health system inputs, namely health service capacity and access (*c*), health expenditure per capita (*e*) and the number of health centres per 100 000 population (*h*), on the likelihood of achieving the universal health service coverage index target of 80% by 2030. The simulation employed the L-BFGS-B optimization algorithm to estimate the percentage increase that would be required for each input factor to achieve a universal health service coverage index of 80% by 2030.^26^ We used initial guesses of 50% increases for all factors, with bounds set between 0% and 100%. The objective function minimized the squared difference between the projected service coverage in 2030 and the target value of 80%. Specifically, the model projected the effect of doubling the value of each input factor individually and in combination, thereby enabling the potential contribution of each factor to the overall universal health service coverage index to be analysed in detail. A Bayesian simulation approach was implemented, in which changes in these input factors were simulated to identify the percentage increase in each factor required to meet the 80% target.

The simulation used a logistic regression model with the form:
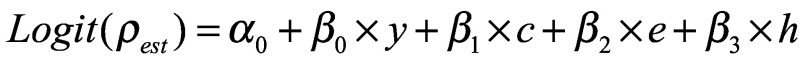
(1)where *p_est_* is the estimated probability of achieving a specific value, *α0* represents the intercept and *β0, β1, β2* and *β3* are the coefficients for the year (*y*), *c*, *e* and *h*, respectively. For the predictive analysis, the model was fitted to historical data and the estimated coefficients were used to project the universal health service coverage index to 2030 under various hypothetical increases in the values of three input factors.

## Results

### Descriptive and trend analysis

**Fig. 1:**
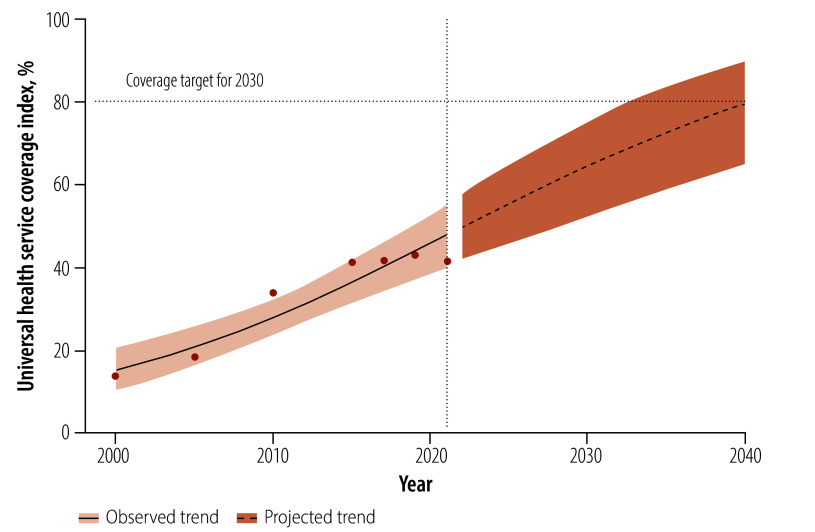
Trend in, and projection of, the universal health service coverage index, Ethiopia, 2000–2040

Note: Observed and projected trend lines were derived using a Bayesian linear regression model. The blue dots indicate observations for 2000, 2005, 2010, 2015, 2017, 2019 and 2021.

Progress towards UHC in Ethiopia has been characterized by early gains and recent stagnation: the universal health service coverage index increased steadily from 2000 to 2015, then more slowly between 2015 and 2019 (Fig. 1). Between 2019 and 2021, progress stalled entirely. Consequently, year-on-year improvements have been limited in recent periods. Moreover, this decline affected all subindices.

Table 2 shows that, among subindices, the infectious disease subindex increased most between 2000 and 2020: the annual percentage change in service coverage increased by an average of 10.7% (95% CrI: 6.5–15.2), which represents a rapid improvement from a very low starting point. There were also steady improvements in the composite universal health service coverage index and the reproductive, maternal, neonatal and child health subindex, which demonstrated average annual percentage changes in coverage of 5.7% (95% CrI: 3.2–8.3) and 5.3% (95% CrI: 3.3–7.5), respectively. By contrast, the service capacity and access and noncommunicable disease subindices increased more slowly: the average annual percentage change was 2.9% (95% CrI: 2.3–3.3) and 1.4% (95% CrI: 1.0–1.8), respectively.

**Table 2:** Observed and predicted health service coverage and probability of achieving 80% coverage by 2030, Ethiopia, 2000–2030

Health service coverage parameter	% (95% CrI)					
	Coverage^a^				Average annual change between 2000 and 2020	Probability of reaching the target of 80% coverage by 2030
	2000	2010	2020	2030		
Reproductive, maternal, neonatal and child health subindex	18.7 (13.9–24.1)	32.6 (28.6–36.8)	52.6 (46.3–58.6)	68.4 (59.1–77.4)	5.3 (3.3–7.5)	1.4 (1.4–2.9)
Infectious disease subindex	4.8 (2.8–7.9)	14.5 (11.0–18.2)	36.8 (28.0–46.4)	66.1 (48.0–80.1)	10.7 (6.5–15.2)	2.8 (2.3–3.7)
Noncommunicable disease subindex	40.0 (37.8–41.8)	46.2 (45.1–47.1)	52.4 (51.2–53.7)	58.5 (56.3–61.3)	1.4 (1.0–1.8)	0 (0–0)
Service capacity and access subindex	12.0 (9.0–16.0)	13.5 (10.0–17.0)	21.0 (17.0–25.5)	28.0 (20.0–35.6)	2.9 (2.3–3.3)	0 (0–0)
Composite universal health service coverage index	15.3 (10.7–21.0)	28.0 (23.9–32.6)	45.9 (39.2–52.6)	64.7 (52.6–75.2)	5.7 (3.2–8.3)	0.4 (0.3–1.0)

CrI: credible interval.

^a^ Coverage was derived using a Bayesian linear regression model.

**Fig. 2:**
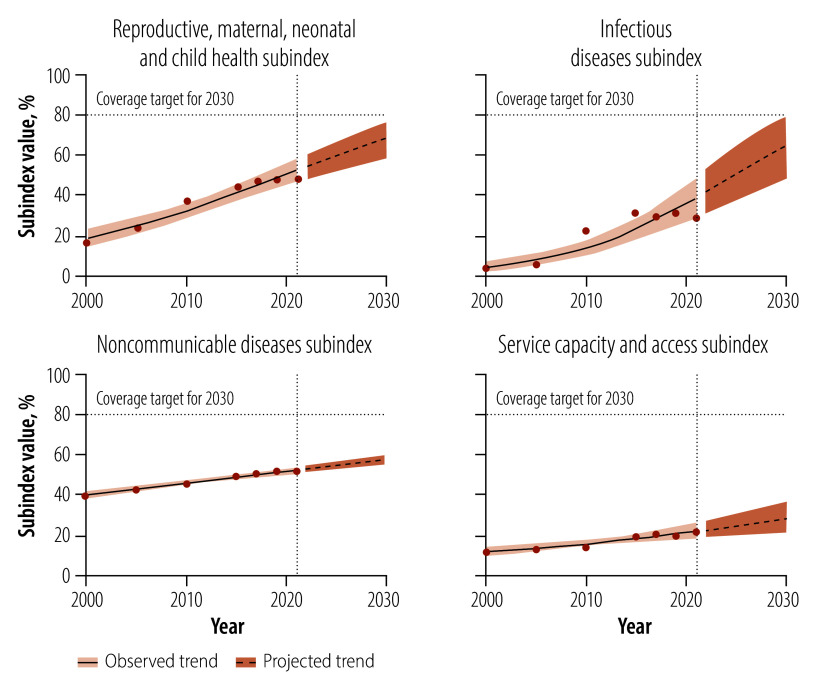
Trends in, and projections of, universal health service coverage subindices, Ethiopia, 2000–2030

Note: The universal health service coverage index is derived from the reproductive, maternal, neonatal and child health subindex; the infectious diseases subindex; the noncommunicable disease subindex; and the health service capacity and access subindex. Observed and projected trend lines were derived using a Bayesian linear regression model. The orange dots indicate observations for 2000, 2005, 2010, 2015, 2017, 2019 and 2021.

As shown in Fig. 2 and Table 2, progress across UHC subindices was uneven between 2000 and 2020. The projected coverage for 2030 was highest for the reproductive, maternal, neonatal and child health subindex, at 68.4% (95% CrI: 59.1–77.4), followed by the infectious disease subindex (coverage: 66.1%; 95% CrI: 48.0–80.1), the noncommunicable disease subindex (coverage: 58.5%; 95% CrI: 56.3–61.3) and the service capacity and access subindex (coverage: 28.0%; 95% CrI: 20.0–35.6).

**Fig. 3:**
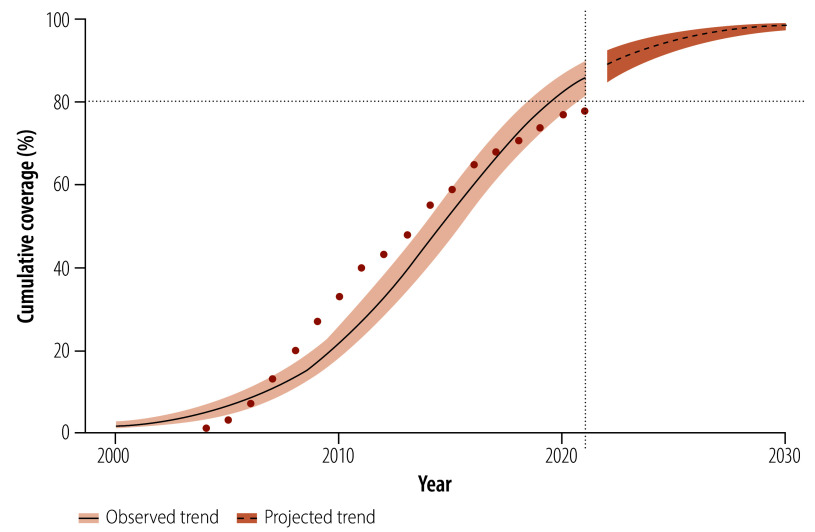
Trend in, and projection of, antiretroviral therapy coverage, Ethiopia, 2000–2030

In contrast, there has been substantial growth in ART coverage since 2000 and the 80% target had almost been achieved by 2020 (Fig. 3). Projections indicate that progress will continue and that coverage will be close to 100% by 2030.

ART: antiretroviral therapy.

Note: Observed and projected trend lines were derived using a Bayesian linear regression model. The orange dots indicate yearly observations between 2005 and 2022.

### Bayesian projection modelling

Fig. 1 and Table 2 indicate that Ethiopia’s universal health service coverage index was projected to reach 64.7% (95% CrI: 52.6–75.2) by 2030 and may reach 80% by 2040. However, as the probability of achieving the 80% target by that year was only 0.4% (95% CrI: 0.3–1.0), it is unlikely to be met. In addition, the probability of achieving the 80% target by 2030 was 1.4% (95% CrI: 1.4–2.9) for the reproductive, maternal, neonatal and child health subindex; 2.8% (95% CrI: 2.3–3.7) for the infectious disease subindex; and 0% (95% CrI: 0.0–0.0) for both the noncommunicable disease and service capacity and access subindices.

Table 2 also reports the uncertainty in index projections for 2030. The uncertainty was greatest for the infectious disease subindex, which had a 95% CrI of 48.0–80.1%. The 95% CrI for the reproductive, maternal, neonatal and child health subindex was 59.1–77.4%, whereas the noncommunicable disease subindex had a narrower 95% CrI of 56.3–61.3%, indicating greater precision. The 95% CrI for the composite universal health service coverage index was 52.6–75.2%, which indicates uncertainty in the progress likely to be made.

### Simulation model

In the simulation model, doubling the level of all three key health system inputs raised the projected universal health service coverage index in 2030 to only 69.6%, compared with the 64.7% predicted by Bayesian projection modelling. In particular, doubling service capacity yielded a 3.4% increase in the universal health service coverage index for 2030, doubling health expenditure per capita yielded a 2.6% increase and doubling the number of health centres per 100 000 population yielded a 5.4% increase. Even with these improvements, the 80% target for universal health service coverage would still not be met by 2030.

## Discussion

Our study investigated the attainability of the universal health service coverage index target of 80% by 2030 globally using Ethiopia as a case study. Historical progress towards achieving the target and future prospects in the country were evaluated through trend analysis, projections and simulation modelling. We found that progress had been made but that substantial challenges persist. There were two distinct phases: the universal health service coverage index increased steadily from 2000 to 2015 but progress had stagnated by 2021. This pattern reflects the importance of not only initiating health reforms but also continuously adapting and reinforcing them to ensure durable progress.

Our trend analysis highlighted a clear shift from steady gains to stagnation. Ethiopia’s initial UHC gains were largely due to its primary health care approach, which involved expanding access to health services to most of the remote population groups.^13^^,^^17^ The slowdown in UHC improvements after 2015 underscores the vulnerability of health system gains to external shocks, such as the COVID-19 pandemic, and the fragility of service delivery mechanisms. Moreover, the uneven progress across health service coverage subindices we observed reveals persistent disparities in health services: while maternal, child and infectious disease services continued to improve, noncommunicable disease coverage remained limited, which reflects the historical prioritization of communicable disease control by Ethiopia’s health system. The country’s experience illustrates the importance of building resilient and balanced health systems capable of sustaining gains across service domains during crises. Resource use can be optimized and service delivery can be enhanced by adopting a system-wide, integrated approach that combines the prevention and treatment of infectious diseases with maternal and child health initiatives and the management of noncommunicable disease.^27^ This holistic strategy can help mitigate service overlaps and redundancies while addressing the interlinked nature of many health problems.^28^^,^^29^

In contrast to the overall stagnation of progress on universal health service coverage, Ethiopia achieved notable success in scaling up ART. The country is predicted to surpass the 80% ART coverage threshold well ahead of 2030. This achievement is due to strong government commitment, coordinated efforts, advocacy, community mobilization, global solidarity, health systems strengthening and a public health approach to ART delivery.^30^ Political commitment was central to the country’s remarkable progress.^31^ Similarly, establishing a strong political will around UHC is essential, with UHC established as a moral, economic and social priority.^32^ Global health organizations, including the Global Fund and the United States’ President's Emergency Plan for AIDS Relief,^33^^,^^34^ have contributed hugely to the success of scaling up ART in Ethiopia. In addition, the so-called diagonal approach, whereby disease-specific initiatives are leveraged to strengthen the broader health system, has helped the country scale up ART.^31^ The lessons learnt from scaling up ART could be applied in accelerating progress towards UHC.^32^

Our projection modelling indicates that the probability Ethiopia will achieve the 80% universal health service coverage target by 2030 is only 0.4%. The 95% credible interval we report captures the best- and worst-case scenarios, which could offer policy-makers valuable evidence for preparedness, planning and resource allocation. Other low- and middle-income countries could also use such predictive tools to identify health service gaps, to anticipate trajectories in coverage and to prioritize targeted strategies that will accelerate progress on UHC. In addition, our simulation model demonstrated that simply expanding health system inputs, such as funding, facilities or workforce, yields only a modest increase in the universal health service coverage index, even under an optimistic scenario. This finding indicates that structural inefficiencies may prevent resources from being translated effectively into improved service delivery. The lesson for Ethiopia and comparable low- and middle-income countries is clear: resource growth alone is insufficient.^35^ Systemic reforms are vital, both within the health system itself but also outside it, in the broader socioeconomic and political context.^9^^,^^36^

Our study demonstrates that, by combining projections and scenario simulations, Bayesian modelling offers a powerful, flexible, decision-support tool.^37^ The model enables policy-makers to evaluate trade-offs, estimate timelines and identify priority areas. Moreover, a study conducted in Bangladesh shows that Bayesian linear regression with noninformative priors and Markov Chain Monte Carlo simulations can produce probabilistic forecasts, credible intervals and assessments of inequality.^23^ Such analytical tools can support strategic planning by estimating service coverage under various investment and policy scenarios.^38^ As demonstrated in our study, these models go beyond monitoring, they facilitate data-driven planning, prioritization and greater transparency in developing policies on UHC. Our model has been used in other studies, for example in Bangladesh and Ghana.^23^^,^^39^

Ethiopia’s experience raises important questions about the feasibility of achieving the 80% universal health service coverage target by 2030 globally.^40^ The country’s initial progress, followed by stagnation, illustrates the difficulty of sustaining gains without systemic reforms and building resilience.^41^^,^^42^ In contrast, countries such as Australia and Thailand have already achieved UHC thanks to greater institutional capacity and stronger long-term investment.^2^^,^^43^^–^^45^ The question arises, then, of whether it is appropriate to apply a uniform global benchmark to countries with vastly different health-system capacities, resources and starting points.^46^^,^^47^ A more flexible, context-specific approach to setting UHC goals is needed: one that prioritizes incremental progress, equity and system resilience over rigid numerical thresholds.^48^ In addition, national policy-makers and their global health partners should recognize that achieving UHC is a long-term, context-specific and adaptive process. Countries need support to set realistic, nationally appropriate targets and to build the institutional capacity required to respond to changing health demands and external shocks.^49^^,^^50^

Our study has some limitations. First, the study relies on historical data from 2000 to 2021 and the assumptions made in the Bayesian model could introduce potential inaccuracies. Furthermore, our projections fail to consider recent crises, including internal conflicts, which may negatively impact Ethiopia's progress towards UHC. Additionally, the model overlooks the social determinants of UHC and does not include financial risk protection. Consequently, the study’s findings should be interpreted with caution.

In conclusion, our study investigated the attainability of the universal health service coverage target of 80% by 2030 globally using Ethiopia as a case study. Although the country made steady progress between 2000 and 2015, coverage has since stagnated, and our projections suggest that the 2030 target is unlikely to be met. Simulation modelling showed that expanding health system inputs alone produced only marginal gains, highlighting the need for structural reforms and improved governance. Ethiopia’s success in scaling up ART demonstrates that political commitment, community engagement and international support can be transformative. Modelling tools, such as those used in our study, can help low- and middle-income countries anticipate challenges, prioritize resources and design context-specific pathways towards UHC. Achieving UHC requires flexible benchmarks that reflect national context and prioritize equity, resilience and incremental progress. 
